# Gender-specific associations between *ADIPOQ* gene polymorphisms and adiponectin levels and obesity in the Jackson Heart Study cohort

**DOI:** 10.1186/s12881-015-0214-x

**Published:** 2015-08-20

**Authors:** Pia Riestra, Samson Y. Gebreab, Ruihua Xu, Rumana J. Khan, Aurelian Bidulescu, Adolfo Correa, Fasil Tekola-Ayele, Sharon K. Davis

**Affiliations:** 1 National Human Genome Research Institute, Genomics of Metabolic, Cardiovascular and Inflammatory Disease Branch, Social Epidemiology Research Unit, National Institutes of Health, 10 Center Drive, Bethesda, MD 20892 USA; 2 School of Public Health, Indiana University Bloomington, 1025 E. 7th Street, Suite 111, Bloomington, IN 47405 USA; 3 Jackson Heart Study, Jackson Medical Mall, 350 West Woodrow Wilson Av., Suite 701, Jackson, MS 39217 USA; 4 National Human Genome Research Institute, Center for Research on Genomics and Global Health, National Institutes of Health, 12 South Drive, Bethesda, MD 20892 USA

## Abstract

**Background:**

Despite the important role of adiponectin in regulating general metabolic homeostasis, analysis of genetic determinants of adiponectin and the related cardio-metabolic traits in African American population has been limited and inconsistent.

Considering the high genetic admixture of African Americans and thus the important population stratification that may confound the genetic-trait associations, the objective of this work was to perform a comprehensive analysis of the associations between *ADIPOQ* variants and adiponectin levels and obesity phenotypes in a large African American population from the Jackson Heart Study (JHS) cohort.

**Methods:**

Genotype data was available for 2968 JHS participants (1131men; 1837women). Single Nucleotide Polymorphisms (SNPs) were selected by a Tag-SNP Approach and literature review. The genotype imputation was performed using IMPUTE2 software and reference phased data from the 1000G project. PLINK software was used for the genetic analysis. Plasma specimens were analyzed by ELISA for adiponectin levels. All analyses were controlled for population stratification assessed by Individual Proportions of European Ancestry (PEA) estimates calculated in HAPMIX using ancestry informative markers (AIMs).

**Results:**

We found a gender-dependent association of some *ADIPOQ* variants and adiponectin levels. In women four of the studied polymorphisms (rs6444174, rs16861205, rs1403697, rs7641507) were associated with adiponectin levels after Bonferroni correction and controlling for the percentage of PEA, age, annual household income and smoking. These results were consistent with the haplotype analysis. The association between the rs12495941 variant and obesity is modulated by the PEA, so that the relationship between the G allele and a higher incidence of obesity was present in those individuals within the lower PEA group. In addition we found an effect modification of obesity on the association between the *ADIPOQ* rs6444174 SNP and BMI so that the presence of the T allele was negatively and significantly associated with BMI only in participants with a normal weight.

**Conclusions:**

In this large African American cohort, *ADIPOQ* variants were associated with adiponectin levels in a gender-dependent manner and the relationship of some of these variants with obesity and BMI was modulated by the PEA and obesity status respectively. This suggests that the effects of these polymorphisms on adiponectin and obesity phenotypes are subject to a strong interaction with genetic and environmental factors in African American population.

**Electronic supplementary material:**

The online version of this article (doi:10.1186/s12881-015-0214-x) contains supplementary material, which is available to authorized users.

## Background

In the last decade obesity has reached epidemic proportions affecting one-third of the adult U.S. population and is considered a major risk factor for development of Type 2 Diabetes Mellitus (DM2), hyperlipidemia and hypertension. All these disorders cluster together in what we know today as the Metabolic Syndrome. However, in the U.S the obesity epidemic disproportionately affects certain racial/ethnic minorities and it is generally known that the prevalence of obesity varies widely by ancestry, especially in African American women who also display a more severe metabolic profile related to the presence of obesity [1].

Adiponectin is an adipose tissue-specific hormone that is responsible for increasing energy expenditure and lipid catabolism as well as enhancing fatty acid oxidation and insulin sensitivity [2]. In contrast to most other adipocytokines, adiponectin appears in decreased concentrations in obese subjects [3]. Consequently, it is considered to be a protective factor for cardio metabolic alterations [4] and has been proposed as a potential biomarker for therapeutic intervention for obesity, diabetes, and other cardiovascular disorders [5]. African Americans present lower serum adiponectin levels independent of their body mass index (BMI) [6–8] and higher prevalence of obesity with a more severe phenotype related to metabolic alterations compared to Caucasians and other ethnic groups.

Plasma adiponectin levels are highly heritable ranging from 40 to 70 % [9, 10] and have been associated with different genetic loci [11]. However the adiponectin gene (*ADIPOQ*) located at position 3q27, remains the main genetic determinant of plasma adiponectin levels [12]. The *ADIPOQ* gene spans 1.579 kb and contains 3 exons. The translation start point is located in exon 2 [13]. Several single nucleotide polymorphisms (SNPs) located in the *ADIPOQ* gene have been associated with adiponectin serum levels [13], body adiposity [14] and metabolic alterations [15, 16], making this gene a candidate for obesity and metabolic syndrome associated traits. However, a limited number of studies that addressed the study of genetic variants in the *ADIPOQ* gene in relation to adiponectin levels and obesity phenotypes in African Americans [16, 17], yielded conflicting results mainly because of small sample size [17], inclusion of only one gender in the analysis [7], or the confounding effect of an unadjusted population structure [18].

In the present study we tested the genetic association of SNPs in *ADIPOQ* with obesity indexes (BMI, body fat content, waist (WC) and hip circumferences (HC)) and serum adiponectin levels in African Americans from the Jackson Heart Study (JHS) with adjustment for population structure using a dense panel of ancestry informative markers (AIMs).

## Research design and methods

### Study participants

The JHS is a single-site, prospective cohort study of risk factors and causes of heart disease in adult African Americans. A convenience sample of 5,301 self-reported African Americans, aged 21–94 years, residing in three adjacent counties in the Jackson, MS metropolitan area were recruited, interviewed and examined by certified technicians according to standardized protocols at a baseline exam visit (2000–2004) [19, 20]. The clinic visit included physical examination, anthropometry, survey of medical history and of cardiovascular risk factors and collection of blood and urine for biological variables. Two subsequent examinations were conducted in 2005–2008 and 2009–2013. The data for this study includes a total of 2934 unrelated participants (1130 men and 1834 women) who had *ADIPOQ* SNPs genotyping information and adiponectin measurements conducted on serum specimens collected at baseline, and who underwent multi-detector computed tomography (CT) measurements for visceral adiposity between 2007 and 2009 during the second JHS examination (JHS Exam 2). Written consent was obtained from each participant at the inception of the study. The study protocol was approved by the Institutional Review Boards of the National Institutes of Health and the participating JHS institutions_–including the Tougaloo College, the Jackson State University and the University of Mississippi Medical Center.

### Biochemical and anthropometric measurements

Adiponectin measurement was derived from venous blood samples drawn from each participant at baseline visit after at least 8 h of fasting. Vials of serum were stored at the JHS central repository in Minneapolis, MN, at −80 °C until assayed. Adiponectin concentration was measured in 2008–2012 as total plasma adiponectin by an ELISA system (R&D Systems; Minneapolis, MN, USA) [19, 21]. The inter-assay coefficient of variation was 8.8 %. No biological degrading has been described using stored specimens, indicating a high validity for measurement [22].

For anthropometric variables, weight was measured on a balance scale, in light clothing, without shoes, and recorded to the nearest 0.5 kg. BMI was calculated as weight in kilograms divided by height in square meters. Overweight/obesity status was assessed based on BMI: [21] individuals with BMI < 25 kg/m^2^ were considered of normal weight (NW), individuals with BMI ≥ 25 kg/m^2^ and < 30 kg/m^2^ were considered overweight (OW), and those with BMI ≥ 30 kg/m^2^ were considered obese. Waist circumference (WC) was measured at the level of the umbilicus using anthropometric tape; hip circumference (HC) was measured at the level of the widest circumference over the greater trochanter. The measurements were recorded to the nearest centimeter upon the end of exhalation. Body fat composition (expressed as total fat mass (kg), and percent of body fat (%) were assessed using a Tanita (Arlington Heights, IL, USA) TNF 300A impedance body composition analyzer. The research CT protocol for abdominal adipose tissue (VAT) and subcutaneous adipose tissue (SAT) measurements has been reported previously [23]. Briefly, the CT images included scout images, one ECG gated series of the entire heart, and a series through the lower abdomen detected by computed tomography system equipped with cardiac gating (GE Healthcare Lightspeed 16 Pro, Milwaukee, WI, USA). The acquired abdominal imaging slices covering the lower abdomen from L3 to S1 were used for assessing VAT and SAT. Briefly, 24 contiguous 2-mm-thick slices centered on the lumbar disk space at L4–L5 were used for this analysis; 12 images before the center of the L4–L5 disk space and 12 images after the disk space were used for quantification of VAT and SAT. The abdominal muscular wall was first manually traced, and the fat volumes in different compartments were measured by semiautomatic segmentation technique. Volume analysis software (Advantage Windows; GE Healthcare, Waukesha, WI) was used to segment and characterize each individual voxel as a tissue attenuation of fat using a threshold range of −190 to −30 Hounsfield units. The VAT and SAT volumes were the sum of VAT and SAT voxels over 24 slices. Participants were excluded from the CT scan Exam if: 1) body weight was greater than 350 lbs. (~160 kg); 2) pregnant or unknown pregnancy status; 3) female participant < 40 years of age; and 4) Male participant < 35 years of age. SAT was defined as the total fat volume minus the visceral and intramuscular fat volume. A total of 10 participants were excluded based on these criteria.

### Covariates

All covariate variables were collected at baseline and were chosen because of their influence on adiponectin levels and obesity measurements. Age was derived from date- of-birth. Socioeconomic status (SES) was based on self-reported annual household income and divided into three categories (< $19,999, $20,000 – $49,999, > $50,000). Smoking status was defined as current smokers and non-smokers.

The proportion of European ancestry (PEA), an estimate of the global proportion of individual European ancestry for our sample, was calculated using HAPMIX in analyses supported by the CARe consortium [24–26]. Briefly, two datasets were used as reference populations: 1,178 European Americans samples and 756 samples of African ancestry, represented here by the Yoruba (YRI) from West Africa were used as parental samples to select the Ancestry Informative Markers (AIMs) from the Affymetrix 6.0 genome-wide genotyping platform. After removing related pairs and outlier samples determined by quality control procedures using EIGENSOFT, a total of 3,192 unlinked AIMs were obtained with allele frequency between parental samples of at least 30 %. Then, the HAPMIX program was used for inferring local ancestry (0, 1 or 2 European chromosomes) at each location in the genome on the CARe samples, using phased CEU and YRI haplotypes from HapMap3 as reference [24]. HAPMIX was run in a mode that assigns European or African ancestry to each allele, thus resolving the local ancestry of each allele when both genotype and local ancestry were heterozygous. Finally, the proportion of the Global European Ancestry for each sample was computed as the average of local ancestry estimates across the genome (scaled to 0.0, 0.5 or 1.0). The proportion of global European ancestry estimates for this sample study had a median of 15.0 %.

### SNP selection, genotyping and imputation

With the aim to investigate all the genetic variability in *ADIPOQ*, we used a tag SNP approach for the selection of the genetic variants in the study. This tagging approach was applied on the entire set of common genetic variants (including 5 kb upstream of the first exon and 5 kb downstream of the last exon of the gene), with minor allele frequency (MAF) ≥1 % in Yoruba population (YBI) from the International HapMap Project (release #24; http://www.hapmap.org). Tagging SNPs were selected by the Tagger algorithm available through Haploview [27], using a pairwise SNP selection with a minimum r^2^ threshold of 0.8. This process resulted in a selection of 14 tagging SNPs for *ADIPOQ*, with a mean r^2^ of the selected SNP of 0.969. This selection therefore captures a very high degree (over 95 %) of the known common variability in this gene. Additional relevant candidate SNPs were selected after reviewing the literature, therefore providing a basis for comparison with the JHS. For this study a total of 39 SNPs in the *ADIPOQ* gene were selected.

For genotype imputation to infer *ADIPOQ* SNPs genotypes in our study, IMPUTE2 software and reference phased data from the 1000G project http://www.sph.umich.edu/csg/yli/mach/download/1000G.2012-03-14.html) were used. Details regarding the generation of the data can be found in the Phase 1 article (The 1000 Genomes Project Consortium, 2012) [28]. Related individual were removed before the genotype imputation.

### SNPs quality control

SNP-level quality control metrics were applied prior to downstream analyses. These were: call rate ≥ 95 %, minor allele frequency (MAF) ≥ 1 %, Hardy-Weinberg equilibrium (HWE) *p* > 10^−3^, and quality measures for imputed SNPs (r^2^ ≥ 0.3).

Additional file 1: Table S1 shows the characteristics, minor allele frequencies and HWE p-values for the studied *ADIPOQ* SNPs. Of the 34 SNPs we excluded 2 from the analysis due to low imputation quality (r^2^ < 0.3) Seven SNPs were not consistent with HWE (*p* < 0.001) and where removed for the analysis. In this cohort five variants found to be nearly monomorphic with a MAF < 0.04 and thus excluded from further analysis (rs17366743, rs17366568, rs17373414, rs17366499, rs2036373). After the exclusion, a total of 24 SNPs analyzed in the JHS cohort. The minor allele frequencies for the analyzed SNPs ranged from 5 to 45 %.

### Statistical analysis

Statistical Package SAS version 9.3 (SAS Institute Inc., Cary, NC, USA) was used for descriptive statistical analysis. Participant characteristics were summarized with means and standard deviations for continuous variables and counts and percentages for categorical variables.

Power analyses for the tests of association were computed using the QUANTO software (available at http://biostats.usc.edu/software), using the minor allele frequencies and mean values of serum adiponectin levels from the JHS study and the effect sizes originally reported by previous studies [29]. Assuming a *p* value of 0.001 and a power of 80 %, we will require 845 subjects in order to detect a 2 % of variation in adiponectin levels. Hardy-Weinberg deviations for each of the *ADIPOQ* SNPs included in the study were tested by a chi-squared goodness of fit test.

Association analyses between the SNPs and the selected variables were conducted in PLINK [30]. Association between *ADIPOQ* SNPs and continuous (BMI, WC, HC, SAT, VAT) and categorical outcomes (obesity) were tested by linear and logistic regression models under an additive genetic model using age, gender, BMI, SES, and smoking as covariates for model adjustment except in the analysis with BMI. To control for population stratification we include the Percentage of European Ancestry (PEA) as a continuous variable into the models.

Adiponectin and BMI values were log transformed to obtain better approximations of the normal distribution prior to analysis. A Bonferroni correction was applied to the a priori alpha level of 0.05 and was calculated based on the number of individual models examined for each of the SNPs on the adiponectin gene (0.05/24). Therefore, a p-value threshold of 0.002 was used to determine Bonferroni-corrected statistical significance for *ADIPOQ* SNPs.

Pair-wise linkage disequilibrium (LD) and haplotype blocks were assessed by Haploview (Broad Institute, MA, USA) [31]. Haplotypes were analyzed using PLINK association analysis for the identified haplotype blocks using linear regression in PLINK. Haplotypes with estimated frequency < 5 % were excluded from the analysis. Global p-values are obtained by omnibus tests jointly estimating all haplotype effects and linear regression analysis was used for the individual haplotype association.

## Results

### General characteristics of the Jackson Heart Study participants

The main characteristics of the study population disaggregated by gender are presented in Table 1. Of the 2968 self-identified African American participants, 1131 were men and 1837 were women. The presence of overweight and obesity was significantly higher in women compared to men (88.8 % vs. 82.6 %, *p* = 0.001). Compared to men, women had significantly higher BMI (30.0 vs. 33.3, *p* < 0.0001), total body fat content (29.6 % vs. 43.3 %, *p* < 0.0001) and SAT (1721.9 units vs. 2687.5 units, *p* < 0.0001). Women also presented higher adiponectin serum levels than men (6.04 μg/mL vs. 4.08 μg/mL, *p* < 0.0001) and a higher proportion of annual household income < $19,999 (32.13 % vs. 19.11 %), *p* < 0.0001). Compared with women, men had higher VAT (903.7 cm^3^ vs. 948.1 cm^3^, *p* < 0.05) and PEA (17 % vs. 18 %, *p* = 0.006) as well as higher annual household income > $50,000 (46.65 % vs. 28.4 % *p* < 0.0001).

**Table 1:** Clinical and socio-demographic characteristics of the study participants, stratified by gender

Variable	Men (*N* = 1131)	Women (*N* = 1837)	*p*-value
Age (y)	53.75 ± 13.02	54.86 ± 12.73	0.022
Adiponectin (ug/mL)	4.08 ± 3.38	6.04 ± 4.46	< .0001
Height (cm)	177.5 ± 6.88	164 ± 6.50	< .0001
Weight (kg)	94.76 ± 21.70	89.54 ± 21.71	< .0001
BMI (kg/m^2^)	30.01 ± 6.33	33.26 ± 7.79	< .0001
Overweight/Obese	82.65 %	88.88 %	< .0001
(BMI ≥ 25)			
WC (cm)	101.6 ± 15.42	101.1 ± 16.89	0.35
Waist-hip ratio	0.94 ± 0.06	0.87 ± 0.08	< .0001
Total body fat (%)	29.61 ± 7.96	43.26 ± 7.02	< .0001
Fat mass (kg)	64.89 ± 32.12	86.35 ± 34.34	< .0001
Total fat (cm^3^)	2670.0 ± 1051.2	3591.3 ± 1175.7	< .0001
VAT (cm^3^)	948.1 ± 425.3	903.7 ± 390.6	0.037
SAT (cm^3^)	1721.9 ± 794.3	2687.5 ± 964	< .0001
PEA mean (SD)	0.18 ± 0.09	0.17 ± 0.08	0.0068
Smoking (%)	17.94 %	11.07 %	< .0001
Annual household income:			
≤ $19,999	19.11 %	32.13 %	< .0001
$20,000-49,999	34.25 %	39.40 %	< .0001
$50,000 ≥	46.65 %	28.47 %	< .0001

Data presented as mean ± SD. *BMI* body mass index, *WC* waist circumference, *SAT* subcutaneous adipose tissue, *VAT* visceral adipose tissue, *PEA* Individual Proportions of European Ancestry

When comparing the subject characteristics in men and women by obesity status, weight, WC, HC, WHR and total body fat content, VAT and SAT were significantly higher in obese men and women than in non-obese subjects. Adiponectin levels were significantly lower in overweight and obese study participants compared to men and women of normal weight (data not shown).

### Associations between *ADIPOQ* SNPs and adiponectin levels

Four variants were found to be nominally associated after the Bonferroni correction (*P* < 0.002) with plasma adiponectin levels adjusting for age, gender, BMI and PEA (Additional file 1: Table S2) with p-values ranging from 0.000579 to 3.28 × 10^−6^. The C allele of the SNP rs7641507 showed the strongest association with higher adiponectin levels (*p* = 3.28 × 10^−6^). The major alleles T at SNP rs6444174, A at rs1403697, and G at rs16861205 were also significantly associated with increased adiponectin concentrations. The SNPs rs6444174, rs1403697 and rs7641507 were located in the 3′-UTR and the rs16861205 in the intron 1.

Given the significant gender differences in adiponectin levels we also performed a sex stratified analysis to test gender-specific differences in the effect of these SNPs on serum adiponectin concentrations, as shown in Tables 2 and 3. We found strong significant associations between the *ADIPOQ* SNPs rs6444174 (β 0.1617 (0.0402) *p* = 6.15 × 10^−5^), rs1403697 (β 0.1126 (0.0296) *p* = 0.0001) and rs7641507 (β 0.1127 (0.0296) *p* = 0.0001) and a marginal association of rs16861205 (*p* = 0.03) with serum adiponectin levels after controlling for PEA, age, BMI, annual household income and smoking in women (Table 2). However, these associations were no longer significant in men (Table 3).

**Table 2:** Association between *ADIPOQ* SNPs and adiponectin serum levels in women from the Jackson Heart Study cohort

SNPs		Model 1		Model 2		Model 3	
rs	Alleles	β (SE)	*P* value	β (SE)	*P* value	β (SE)	*P* value
rs4632532	T/C	−0.064 (0.028)	0.023	−0.072 (0.03)	0.031	−0.083 (0.036)	0.023
rs6444174	T/C	0.13 (0.030)	0.000011	0.15 (0.037)	0.000018	0.16 (0.040)	0.000061
rs16861194	A/G	0.019 (0.023)	0.40	0.021 (0.028)	0.44	0.025 (0.031)	0.41
rs182052	G/A	0.019 (0.023)	0.42	0.020 (0.028)	0.48	0.022 (0.031)	0.48
rs710445	A/G	0.019 (0.023)	0.40	0.021 (0.028)	0.44	0.025 (0.031)	0.42
rs16861205	G/A	−0.13 (0.036)	0.00031	−0.11 (0.042)	0.0086	−0.096 (0.046)	0.039
rs822394	C/A	−0.064 (0.045)	0.15	−0.057 (0.055)	0.30	−0.035 (0.059)	0.55
rs12495941	G/T	0.13 (0.082)	0.089	0.0090 (0.11)	0.93	−0.072 (0.12)	0.55
rs7649121	A/T	0.045 (0.070)	0.51	0.066 (0.081)	0.41	0.031 (0.090)	0.73
rs7627128	C/A	−0.014 (0.15)	0.92	−0.15 (0.19)	0.42	−0.073 (0.20)	0.72
rs9877202	A/G	−0.049 (0.042)	0.24	−0.039 (0.051)	0.43	−0.024 (0.054)	0.65
rs1501299	G/T	−0.14 (0.049)	0.0043	−0.11 (0.056)	0.042	−0.130 (0.059)	0.029
rs3821799	T/C	0.031 (0.028)	0.27	0.024 (0.033)	0.46	0.039 (0.036)	0.28
rs9842733	A/T	−0.099 (0.11)	0.38	0.044 (0.13)	0.74	0.027 (0.150)	0.85
rs1403697	A/G	0.088 (0.022)	0.000095	0.10 (0.027)	0.00019	0.11 (0.029)	0.00015
rs7641507	C/T	0.088 (0.022)	0.000094	0.10 (0.027)	0.00019	0.11 (0.029)	0.00014
rs1403696	T/C	−0.046 (0.081)	0.56	−0.0047 (0.10)	0.96	−0.098 (0.11)	0.40
rs13085499	T/C	0.093 (0.11)	0.41	0.088 (0.13)	0.51	−0.0023 (0.14)	0.98

Alleles listed as major/minor allele; Model 1: Adjusted for BMI, age, smoking; Model 2: adjusted for age, smoking, ancestry; Model 3: adjusted for age, smoking, ancestry, BMI, annual household income: The results of the association are listed as the Beta (standard error) (β (SE)) with the corresponding *P*-value. β coefficients represent the change in absolute trait values of each additional risk allele

**Table 3:** Association between *ADIPOQ* SNPs and adiponectin serum levels in men from the Jackson Heart Study cohort

SNPs		Model 1		Model 2		Model 3	
rs	Alleles	β (SE)	*P* value	β (SE)	*P* value	β (SE)	*P* value
rs4632532	T/C	−0.048 (0.038)	0.21	−0.054 (0.044)	0.22	−0.041 (0.047)	0.37
rs6444174	T/C	0.034 (0.040)	0.39	0.054 (0.045)	0.22	0.042 (0.048)	0.38
rs16861194	A/G	0.069 (0.032)	0.032	0.080 (0.036)	0.027	0.083 (0.038)	0.029
rs266729	C/G	−0.039 (0.042)	0.35	−0.025 (0.048)	0.59	−0.022 (0.051)	0.67
rs182052	G/A	0.073 (0.032)	0.024	0.083 (0.036)	0.024	0.090 (0.039)	0.020
rs710445	A/G	0.070 (0.032)	0.027	0.082 (0.036)	0.022	0.086 (0.038)	0.024
rs16861205	G/A	−0.084 (0.050)	0.093	−0.147 (0.057)	0.010	−0.11 (0.061)	0.074
rs822394	C/A	−0.075 (0.062)	0.22	−0.126 (0.070)	0.071	−0.109 (0.07)	0.14
rs12495941	A/G	−0.047 (0.13)	0.72	−0.092 (0.15)	0.56	−0.16 (0.16)	0.32
rs7649121	G/T	0.046 (0.090)	0.60	0.082 (0.11)	0.45	0.095 (0.11)	0.39
rs7627128	A/T	−0.17 (0.19)	0.36	−0.15 (0.26)	0.55	−0.37 (0.28)	0.18
rs9877202	C/A	−0.077 (0.057)	0.18	−0.10 (0.063)	0.099	−0.10 (0.067)	0.13
rs1501299	A/G	−0.019 (0.062)	0.76	−0.063 (0.071)	0.37	−0.11 (0.078)	0.15
rs3821799	G/T	0.036 (0.038)	0.34	0.025 (0.043)	0.56	0.023 (0.046)	0.62
rs1403697	C/T	0.084 (0.030)	0.0063	0.097 (0.034)	0.0051	0.105 (0.037)	0.0048
rs7641507	A/T	0.084 (0.030)	0.0063	0.097 (0.034)	0.0051	0.105 (0.037)	0.0048
rs1403696	A/G	0.079 (0.146)	0.58	0.15 (0.16)	0.37	0.071 (0.15)	0.63
rs13085499	C/T	0.278 (0.162)	0.086	0.31 (0.21)	0.13	0.283 (0.21)	0.19

Alleles listed as major/minor allele; Model 1: Adjusted for BMI, age, smoking; Model 2: adjusted for age, smoking, ancestry; Model 3: adjusted for age, smoking, ancestry, BMI, annual household income. The results of the association are listed as the beta (standard error) (β (SE)) with the corresponding *P*-value. β Coefficients represent the change in absolute trait values of each additional risk allele

### Influence of genetic ancestry on the association of *ADIPOQ* SNPs and obesity

We analyzed the relationship between *ADIPOQ* SNPs and obesity in the group (Table 4) and found a significant association between *ADIPOQ* SNP rs12495941 and obesity (OR 2.3737 (0.2661) *p* = 0.001158). However the significance of this relationship was lost following adjustment for PEA suggesting a modulatory effect of PEA on the association between this SNP and obesity. In order to explore the influence of PEA on the association between *ADIPOQ* SNPs and obesity we stratified the analysis by PEA levels expressed in two categories (low vs. high PEA) based on the median value of this variable (15 %). As shown in Table 5 we observed that the association between the G allele and obesity was present in those individuals within the lower PEA group and this p-value almost reached the Bonferroni p-value threshold (*p* = 0.004) after adjusting for gender, age, and smoking.

**Table 4:** Association between *ADIPOQ* SNPs and obesity in the Jackson Heart Study cohort

SNPs		Model 1		Model 2		Model 3		Model 4		Model 5	
rs	Alleles	OR (SE)	*P* value	OR (SE)	*P* value	OR (SE)	*P* value	OR (SE)	*P* value	OR (SE)	*P* value
rs4632532	T/C	1.033(0.10)	0.76	1.12 (0.12)	0.35	1.16 (0.13)	0.27	1.12 (0.12)	0.35	0.99 (0.10)	0.95
rs6444174	T/C	0.83 (0.11)	0.12	0.79 (0.14)	0.10	0.86 (0.15)	0.32	0.77 (0.14)	0.081	0.82 (0.11)	0.11
rs16861194	A/G	0.90 (0.091)	0.27	0.93 (0.10)	0.52	0.89 (0.11)	0.32	0.94 (0.10)	0.57	0.92 (0.090)	0.38
rs182052	G/A	0.90 (0.092)	0.29	0.94 (0.10)	0.60	0.90 (0.11)	0.38	0.95 (0.10)	0.66	0.93 (0.091)	0.43
rs710445	A/G	0.90 (0.091)	0.28	0.93 (0.10)	0.54	0.89 (0.11)	0.34	0.94 (0.10)	0.59	0.92 (0.090)	0.40
rs16861205	G/A	1.16 (0.13)	0.25	1.24 (0.15)	0.15	1.22 (0.17)	0.22	1.25 (0.15)	0.14	1.16 (0.13)	0.25
rs822394	C/A	1.05 (0.17)	0.75	1.10 (0.20)	0.62	1.09 (0.21)	0.60	1.10 (0.20)	0.62	1.04 (0.17)	0.80
rs12495941	G/T	2.37 (0.26)	0.0011	1.95 (0.36)	0.068	1.93 (0.39)	0.095	1.99 (0.37)	0.063	2.60 (0.26)	0.00028
rs7649121	A/T	0.95 (0.26)	0.87	0.93 (0.31)	0.83	1.12 (0.32)	0.70	0.95 (0.31)	0.89	0.95 (0.26)	0.85
rs7627128	C/A	0.38 (0.68)	0.16	1.19 (0.71)	0.80	1.61 (0.71)	0.50	1.18 (0.72)	0.81	0.37 (0.69)	0.16
rs9877202	A/G	1.03 (0.15)	0.82	1.04 (0.18)	0.81	1.01 (0.20)	0.95	1.04 (0.18)	0.81	1.04 (0.15)	0.77
rs1501299	G/T	1.33 (0.17)	0.091	1.35 (0.19)	0.11	1.28 (0.21)	0.25	1.35 (0.19)	0.12	1.27 (0.16)	0.15
rs3821799	T/C	0.88 (0.11)	0.28	0.84 (0.13)	0.20	0.79 (0.144)	0.11	0.83 (0.13)	0.18	0.88 (0.11)	0.28
rs9842733	A/T	1.71 (0.40)	0.18	2.19 (0.45)	0.086	2.32 (0.500)	0.091	2.37 (0.46)	0.060	1.88 (0.40)	0.11
rs1403697	A/G	0.87 (0.086)	0.13	0.88 (0.10)	0.22	0.89 (0.12)	0.3115	0.87 (0.10)	0.19	0.88 (0.085)	0.16
rs7641507	C/T	0.87 (0.086)	0.13	0.88 (0.10)	0.22	0.89 (0.11)	0.3125	0.87 (0.10)	0.19	0.88 (0.085)	0.16
rs1403696	T/C	0.69 (0.38)	0.34	0.64 (0.47)	0.36	0.51 (0.58)	0.2554	0.68 (0.47)	0.43	0.75 (0.38)	0.46
rs13085499	T/C	1.08 (0.42)	0.84	1.26 (0.50)	0.64	1.47 (0.54)	0.4686	1.37 (0.51)	0.53	1.26 (0.42)	0.57

Model 1: no adjustment; Model 2: adjusted for age, smoking; Model 3: adjusted for age, smoking, PEA; Model 4: adjusted for age, smoking, PEA, annual household income, model 5: adjusted for age, smoking, PEA, sex. The results of the association are listed as OR (standard error) (OR (SE)) with the corresponding *P*-value

**Table 5:** Association between the rs12495941 *ADIPOQ* SNP and obesity risk in participants with high and low PEA from the JHS

	High PEA		Low PEA	
	OR (SE)	*P* value	OR (SE)	*P* value
Model 1	0.96 (0.5842)	0.94	−0.044 (0.16)	0.78
Model 2	−0.042 (0.1575)	0.78	4.33 (0.53)	0.0056
Model 3	−0.0050 (0.1487)	0.97	4.71 (0.54)	0.0042

Model 1: no adjustment; Model 2: adjusted for age, smoking, Model 3: adjusted for age, smoking, sex, annual household income. The results of the association are listed as OR (standard error) (OR (SE)) with the corresponding *P*-value

### Associations between *ADIPOQ* SNPs and obesity phenotypes

Regarding the association between *ADIPOQ* polymorphisms and obesity phenotypes, none of the *ADIPOQ* SNPs studied met the Bonferroni p-value thresholds for a significant relationship with any of the adiposity measures (WC, HC, VAT, SAT, TF) and BMI, after controlling for age, smoking, PEA, and sex (data not shown). However as shown in Table 6 in the analysis stratified by obesity categories (< 30 kg/m2 and ≥ 30 kg/m2), the presence of the T allele of *ADIPOQ* rs6444174 was negatively associated with BMI (β = -0.0417 (0.0115) *p* = 0.0003662) but only in those participants with normal weight after adjusting for age, sex, smoking, PEA, and annual household income. This result provided evidence of an effect modification by the obesity status of the association between *ADIPOQ* rs6444174 and BMI. Using a Chi-square test, we compared the genotype distribution of *ADIPOQ* rs6444174 polymorphism in normal-weight with their distributions in overweight/obese participants. No differences in genotype or allelic distributions among obesity categories were observed (data not shown).

**Table 6:** Association between the rs644417*4 ADIPOQ* SNP and BMI in normal weight and overweight or obese men and women from the Jackson Heart Study cohort

	Normal-weight		Overweight/Obese	
	β (SE)	*P* value	β (SE)	*P* value
Model 1	−0.019 (0.0095)	0.036	−0.0048 (0.0078)	0.53
Model 2	−0.020 (0.0095)	0.032	−0.0049 (0.0077)	0.52
Model 3	−0.035 (0.010)	0.0010	−0.0033 (0.0095)	0.72
Model 4	−0.041 (0.011)	0.00028	−0.0048 (0.0078)	0.53
Model 5	−0.034 (0.010)	0.0017	−0.0057 (0.0092)	0.54
Model 6	−0.041 (0.011)	0.00036	0.0021 (0.010)	0.83

Model 1: no adjustment; Model 2: adjusted for age, smoking; Model 3: adjusted for age, smoking, PEA; model 4: adjusted for age, smoking, PEA, annual household income, model 5: adjusted for age, smoking, PEA, sex. Model 6: Adjusted for sex age, gender, smoking, annual household income and PEA. The results of the association are listed as the beta (standard error) (β (SE)) with the corresponding -value. β Coefficients represent the change in absolute traits values of each additional risk allele

### Associations of common haplotype with adiponectin levels and obesity

**Fig. 1:**
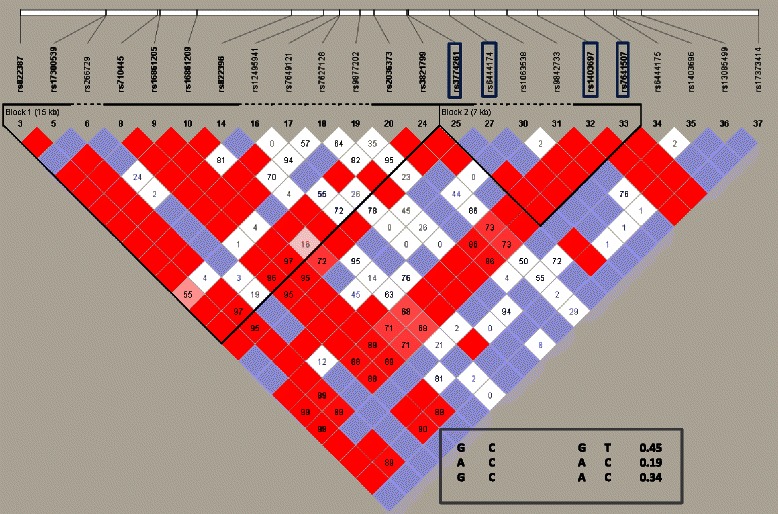
Linkage disequilibrium plot for SNPs in the *ADIPOQ* gene. The value within each diamond is R^2^ between pairs of SNPs. The red to white gradient reflects higher to lower linkage disequilibrium values (red, high; white, low). Most common haplotype blocks codes and their frequencies in women are indicated

The linkage disequilibrium plot for the *ADIPOQ* variants selected in this study is presented in Fig. 1. Two haplotype blocks were identified in *ADIPOQ* among African Americans from the JHS cohort.

In order to study the combined effect of the *ADIPOQ* SNPs in the present study, we tested the association of those SNPs that were significantly associated with adiponectin levels. The four SNPs significantly associated with adiponectin levels in woman are located in the same LD block (Fig. 1). Considering all of the four SNPs simultaneously and using age, sex and BMI and PEA as covariates the haplotype analysis predicted three common haplotypes (frequency > 5 %). The most probable haplotype (CGGT) had an estimated global frequency of 44 %. To assess the association with adiponectin levels, we performed a global test of association across all haplotypes, as well as haplotype-specific association by linear regression analysis.

As shown in Table 7, the haplotype frequencies were not different in men and women for the three mayor haplotype combinations (CGGT/CAAC/CGAC). The haplotype analysis revealed significant associations with adiponectin levels for the four SNPs haploblock CAAC in women but not in men (global *p* = 0.0006 vs. 0.026). After adjustment for PEA, annual household income, BMI and smoking these results were consistent with the gender effect observed in the individual SNP analyses. The haplotype CGGT was associated with increased adiponectin levels (0.00029) while the CGAC haplotype showed the opposite effect (0.001).

**Table 7:** Association between the most common haplotype combinations in the *ADIPOQ* gene and adiponectin levels

	Men				Women			
Haplotype	F	*P* value* (individual)	*P* value (global)	*P* value* (global)	F	*P* value* (individual)	*p*	P*
2222 CGGT	0.477	0.0114			0.45	0.00029		
2111 CAAC	0.207	0.627	0.044	0.026	0.197	0.513	0.000258	0.000674
2211 CGAC	0.341	0.0245			0.348	0.001		

Linear regression analysis was used for the individual haplotype association test. Global P values refer to the global test of association across all detected haplotypes. *p*-value*: adjusted for PEA, annual household income, smoking, BMI and age

## Discussion

In the present study, we examined the relationship between *ADIPOQ* polymorphisms and adiponectin levels and obesity phenotypes in African American participants from the JHS. The *ADIPOQ* gene has been found to be strongly associated with obesity and serum adiponectin levels among numerous study populations [2, 3, 10, 12, 15, 32, 33]. Our study attempted to confirm some of these findings. We evaluated whether *ADIPOQ* SNPs are associated with adiponectin levels and obesity phenotypes in a large population sample of African American men and women. We analyzed a high number of *ADIPOQ* SNPs, some of which have been studied in other ethnic groups [34, 35], in order to capture almost all the known common variability in this gene.

There is growing evidence that *ADIPOQ* variants are related to adiponectin levels and adiposity phenotypes. *ADIPOQ* SNPs genotype frequencies and LD patterns vary widely among ethnic groups and may explain in part the differences in obesity prevalence between non-Hispanic White and African Americans in the U.S. However, SNPs in the *ADIPOQ* gene, have been almost exclusively examined in relation to these variables in European-derived or Asian populations and the few studies conducted in African Americans were small [36] or did not control for population structure using Ancestry Informative Markers (AIMS) as an accurate measure of population stratification.

In this study we found strong novel associations between four *ADIPOQ* polymorphisms (rs6444174, rs16861205, rs1403697, and rs7641507) and adiponectin levels in African American women that were consistent with the haplotype analysis. These associations remained significant after adjustment for PEA, age, BMI, annual household income and smoking. We also found that the *ADIPOQ* rs6444174 variant was associated with BMI among normal weight subjects. In addition, we observed that the association between the rs12495941 and obesity risk appears to be modulated by PEA, so that the association between the G allele and a higher risk of obesity was present in those individuals within the lower PEA.

### Serum Adiponectin levels and *ADIPOQ* gene single nucleotide polymorphisms

We found that the *ADIPOQ* variants rs6444174, rs16861205, rs1403697, and rs7641507 were strongly associated with baseline serum adiponectin levels in a gender dependent manner. Our results showed that the association of *ADIPOQ* polymorphisms with adiponectin levels only remained significant in woman. To the best of our knowledge, the wide sample size of the JHS cohort, permitted for the first time, an examination of this association using a gender stratified analysis in African Americans. These results are consistent with prior GWAS studies that pinpointed the *ADIPOQ* locus as the primary signal for plasma adiponectin levels [12, 37], thus likely the primary source of common variants influencing adiponectin level variations in different populations [13].

Regarding the *ADIPOQ* SNP rs6444174, we detected a novel and significant sex specific association of this polymorphism with serum adiponectin levels. We also found that this *ADIPOQ* variant appears to be related to BMI only in normal weight subjects, and this association was not significant in obese or overweight subjects suggesting an obesity-gene interaction. However, we did not find any direct association of this SNP with other obesity phenotypes. Interestingly based on the ALlele FREquency Database (ALFRED http://alfred.med.yale.edu/.) the ancestral (T) allele of the rs6444174 SNP predominates in European populations where it is almost fixed (100 %), whereas the derived (C) allele is exclusive to African populations (98.7–100 %). Several studies have reported a significant association between BMI and different *ADIPOQ* SNPs [38–40]. However, much of this literature has been inconclusive while other studies have failed to find any associations [7, 41, 42]. To our knowledge, hardly any data exists in the literature regarding the influence of this particular *ADIPOQ* SNP on adiponectin levels or obesity phenotypes. A recent study conducted in Saudi population showed this SNP to be associated only with low HDL-C serum levels. Although the authors did not report the rare allele frequency for this polymorphism in their population [43], demographic data from Saudi Arabia shows 10 % of Afro-Asian ancestry which could explain the presence of this allele in this ethnic group. We hypothesized that this African ancestry-specific allele might be conferring a metabolic protection through higher adiponectin levels and an increased homeostasis of fat deposition in subjects within a normal weight range but that protection or metabolic advantage is susceptible to modification under an obese environment. A study conducted by Rokholm et al [44] found that genes related to body fatness are expressed in a detrimental manner under obesogenic environments. Until now the focus has primarily been on physical activity with the fat and carbohydrate intake as the main modifiers of the genetic effect on adiposity [33, 45]. However, the presence of obesity now represents the main effect modifier. Although this polymorphism is located in the 3′-UTRT non-coding gene region, we postulated that the underlying biology behind the signal was likely to impact gene expression through some obesity mediated regulatory mechanism.

For the rs16861205 *ADIPOQ* SNP variant, we found that the A allele of this SNP was positively correlated with adiponectin levels. However, in this case, the association did not reach the Bonferroni significance threshold in the gender-stratified analysis. To date only two studies have described similar associations. In one of the studies conducted in a Finnish cohort [46], the authors reported an association between the A allele and higher adiponectin levels and lower basal body weight while the other study conducted in Chinese population reported that the AA genotype acts as a protective factor against metabolic syndrome [47]. This polymorphism is an intronic variant, although introns are non-coding regions. There is evidence that intron-derived miRNAs can suppress intracellular RNA homologues and regulate the gene function by repressing translation or cleaving RNA transcripts [48]. Another possibility is that this SNP, would be a marker in linkage disequilibrium with a functional variant.

When analyzing the rs1403697 *ADIPOQ* polymorphism we also found a strong sex-specific association between the major allele A and adiponectin levels. Women carriers of the A allele presented significantly higher plasma adiponectin concentrations. To our knowledge this represents a novel finding because none of the previous studies conducted in different populations including African Americans have found an association between this polymorphism and adiponectin levels [17, 49].

In this study we also identified a novel association between the *ADIPOQ* rs7641507 polymorphism and adiponectin levels. The frequency of the minor allele in our population was 5 %, similar to what is described for YRB HAPMAP population but, interestingly, this polymorphism appears to be fixed in JPT CHB and CEU HAPMAP populations. There is almost no literature regarding the association between this polymorphism and adiponectin levels probably because of its low frequency in many ethnic groups. As an example, this variant was also found to be monomorphic in Hispanics from the IRAS Family study but not in African Americans [17].

With respect to the haplotype analysis, we found a co-existence of two haplotypes with opposite genetic effects in our population that is unique. In accordance with the genotype analyses, the haplotype consisting of the four major alleles CGGT was positively associated with increased serum adiponectin levels.

In summary, the *ADIPOQ* SNPs found to be associated with adiponectin levels in women in this African American cohort differ from those *ADIPOQ* SNPs that have been historically associated with adiponectin levels in other populations. Examples include SNPs such as theG276T SNP in intron 2 and the T45G in exon 2 [50] and other *ADIPOQ* SNPs located at the promoter region and linked to a regulation of the promoter activity [51, 52] [53], as well from those SNPs reported in the scarce studies conducted in African-American population [7, 16, 17, 34, 49] where only the association between the promoter *ADIPOQ* variant rs17300539 and adiponectin levels was consistent with other studies conducted in individuals of European descent [17, 52]. This inconsistency may be the result of the inclusion of participants with different ethnic backgrounds, lack of gender stratified analysis, control for population structure and smaller sample sizes.

This study is a good example of the important role for sex-differentiated effects in the architecture of complex traits. Gender can be considered a measured environmental risk factor which incorporates established anatomical, physiological, and behavioral differences between males and females [54]. The sexual dimorphism in adiponectin levels is well stablished starting at puberty possibly influenced by sexual hormones. The assessment of the influence of sexual hormones and menopause status on the relationship between *ADIPOQ* SNPs and adiponectin concentrations, as well as the possible hormone-SNP interactions which may explain the observed gender differences in adiponectin levels was beyond the scope of the current study. This relationship needs to be further evaluated in future studies in African American who present lower adiponectin levels compared to other ethnic groups.

### Obesity phenotypes and ADIPOQ gene single nucleotide polymorphisms

We found a statistically significant influence of PEA on the association of the *ADIPOQ* rs12495941 SNP and obesity. To the best of our knowledge, this is the first time that a significant effect modification between ethnicity and this *ADIPOQ* variant in determining obesity risk is reported. In our population this *ADIPOQ* variant was associated with an increased obesity risk but only in subjects with a lower PEA or a higher percentage of African ancestry. This finding is in line with a recent study in the same African American cohort where the authors found that the global proportion of European ancestry was directly associated with adiponectin levels only in normal weight and non-diabetic subjects. This suggest an interplay between genetic and environmental factors determining adiponectin levels where PEA acts as a protective factor which may explain ethnic differences in obesity-related metabolic risk [55]. The studies analyzing the association between this polymorphism and adiponectin levels and obesity phenotypes are scarce and yielded disparate results. In Hispanics from the IRAS study, this polymorphism was associated to fasting glucose and SAT but not in African Americans probably due to a sample size of only 560 subjects [17]. The minor allele of this SNP was also strongly associated with higher adiponectin levels in Chinese participants from the Hong Kong Cardiovascular Risk Factor Prevalence Study (CRISPS) [56]. Also in Chinese population a significant association of this variant and adiponectin levels has been reported [57]. However the Framingham Heart Study did not find any association between this SNP and plasma adiponectin levels [52]. Although this *ADIPOQ* variant is a silent polymorphism located in intron 1, and based on reports from the Alibaba 2.1 program, it is not involved in any putative transcription binding site [58]. It has recently been suggested that some organisms possess the ability to selectively use certain codons for transcriptional selection or translational and RNA stability [43], conferring this SNP a potential regulatory activity.

Our research presents some strengths and limitations. The fact that the sample size far exceeds previous studies, permitted us to capture most of the variation across the adiponectin gene in African Americans through a tag-SNP approach rather than a small candidate SNP selection. This is especially relevant to capture specific association signals, particularly in populations with low levels of LD like African Americans. In addition, we have corrected the population stratification with an exhaustive control for genetic ancestry based on AIMs. On the other hand, besides the cross-sectional design of our study, we did not incorporate food and nutrient intake, physical activity, and other environmental covariates. In addition, rare *ADIPOQ* variants are increasingly found to be associated with serum adiponectin levels and have previously been identified as contributors to adiponectin variations in African Americans [17]. We did not include these variants in our report.

It will be necessary to replicate these findings in other unrelated, large and well characterized populations using correct accounting for the genetic ancestry effects.

## Conclusions

In summary, by using a tag-SNP approach we have comprehensively analyzed the association of genetic variants and haplotypes of *ADIPOQ* with adiponectin levels and obesity phenotypes in a large and well-characterized phenotypically African American cohort. We found that variants in the *ADIPOQ* gene are associated with adiponectin levels in a gender dependent way. We also observed that the association between *ADIPOQ* variants and obesity risk and BMI seem to be modulated by other factors such as PEA and obesity status, which suggests that the effects of these polymorphisms are subject to a strong interaction with genetic and environmental factors in the African American population. Interestingly three of the variants related to increased adiponectin levels were monomorphic or rare in other populations conferring this variant’s a potential cardio-metabolic protection in subjects with an African ancestry descent. These findings indicate that African Americans may truly have a unique genetic architecture of adiponectin and obesity phenotypes related to the *ADIPOQ* gene. Moreover, we detected variants and haplotypes for the same gene modulating adiponectin levels in an antithetic fashion, with some related to an increased serum adiponectin and others related to decreased serum levels. This phenomenon points to the complexity of polygenic traits in which multiple alleles underlie the queried phenotype and allele-specific effects contribute to both ends of the phenotype. These observations, in conjunction with sex-specific haplotype effects, highlight the challenge of elucidating the genetic basis of polygenic diseases such as obesity.

## Additional file

Additional file 1: **Table S1.** Characteristics of the selected single nucleotide polymorphisms (SNPs) in the ADIPOQ gene. **Table S2.** Association between ADIPOQ SNPs and adiponectin serum levels in men and women from the Jackson Heart Study cohort. **Table S3.** Association between ADIPOQ SNPs and BMI in normal weight and overweight or obese men and women from the Jackson Heart Study cohort. (DOCX 28 kb)

## Abbreviations

AIMs: Ancestry informative markers
BMI: Body mass index
SNP: Single nucleotide polymorphism
WC: Waist circumference
TF: Total body fat
HC: Hip circumference
WHR: Waist hip ratio
VAT: Visceral adipose tissue
SAT: Subcutaneous adipose tissue
PEA: Individual Proportions of European Ancestry
MAF: Minor allele frequency
