# Supplementary material for: Gender-specific associations between ADIPOQ gene polymorphisms and adiponectin levels and obesity in the Jackson Heart Study cohort
Source: BMC Med Genet. 2015 Aug 20;16:65. doi: 10.1186/s12881-015-0214-x (PMC4593213; doi:10.1186/s12881-015-0214-x)
Supplement: Additional file 1: — Table S1. Characteristics of the selected single nucleotide polymorphisms (SNPs) in the ADIPOQ gene. Table S2. Association between ADIPOQ SNPs and adiponectin serum levels in men and women from the Jackson Heart Study cohort. Table S3. Association between ADIPOQ SNPs and BMI in normal weight and overweight or obese men and women from the Jackson Heart Study cohort. (DOCX 28 kb) [file 12881_2015_214_MOESM1_ESM.docx]

S1: Characteristics of the selected single nucleotide polymorphisms (SNPs) in the *ADIPOQ* gene.

| ***ADIPOQ* SNPs** | **Other Name in the Literature** | **Location on Chromosome 3 (NCBI 35)^(a)^** | **Relation to Gene** | **r^2 (b)^** | **HWE**  **P- value** | **Major Allele** | **Minor Allele** | **MAF** |
| --- | --- | --- | --- | --- | --- | --- | --- | --- |
| **rs6810075** |  | 186548565 | 5’ | 0.84349 | 0.0007 | T | C | 0.34673 |
| **rs4632532** |  | 186551682 | 5’ | 0.8266 | 0 | T | C | 0.45835 |
| **rs6444174** |  | 186573189 | 3'-UTR | 0.99594 | 0.027 | T | C | 0.15706 |
| **rs822387** | T > C | 186556037 | 5'-end | 0.75169 | 0.9854 | T | C | 0.28067 |
| **rs16861194** | −11426 G>A | 186559425 | 5'-end | 0.90636 | 0.0016 | A | G | 0.23027 |
| **rs17300539** |  | 186559460 | 5'-end | 0.45737 | 0.9487 | G | A | 0.02329 |
| **rs266729** | −11377 G>C | 186559474 | 5'-end | 0.86505 | 0.0004 | C | G | 0.14966 |
| **rs182052** | −10068 G>A | 186560782 | Intron 1 | 0.99137 | 0.0012 | G | A | 0.37142 |
| **rs710445** |  | 186561518 | Intron 1 | 0.99253 | 0.0016 | A | G | 0.4029 |
| **rs16861205** |  | 186561634 | Intron 1 | 0.99357 | 0.0312 | G | A | 0.21245 |
| **rs16861209** | −7734 C > A | 186563114 | Intron 1 | 0.64175 | NA | C | A | 0.13884 |
| **rs822391** |  | 186563803 | Intron 1 | 0.67822 | NA | T | C | 0.05145 |
| **rs16861210** |  | 186566498 | Intron 1 | 0.62819 | 0.0003 | G | A | 0.14278 |
| **rs822394** |  | 186566728 | Intron 1 | 0.64793 | 0.913 | C | A | 0.04868 |
| **rs822396** | -3964 A>G | 186566877 | Intron 1 | 0.67822 | NA | A | G | 0.18764 |
| **rs17366499** |  | 186567074 | Intron 1 | 0.02666 | 0.5763 | A | G | 0.00071 |
| **rs12495941** | G > T | 186568180 | Intron 1 | 0.61244 | 0.3807 | G | T | 0.35333 |
| **rs7649121** |  | 186568785 | Intron 1 | 0.6838 | 0.6372 | A | T | 0.15217 |
| **rs7627128** |  | 186568799 | Intron 1 | 0.68362 | 0.7607 | C | A | 0.15217 |
| **rs9877202** |  | 186569607 | Intron 1 | 0.71281 | 0.697 | A | G | 0.13273 |
| **rs2036373** |  | 186570191 | Intron 1 | 0.98257 | 0.9634 | T | G | 0.06391 |
| **rs17366568** |  | 186570453 | Intron 1 | 0.97341 | NA | G | A | 0.02249 |
| **rs2241766** | +45T>G | 186570892 | Exon 2 | 0.96694 | NA | T | G | 0.0465 |
| **rs1501299** | G276T | 186571123 | Intron 2 | 0.98519 | 0.5123 | G | T | 0.35484 |
| **rs3821799** |  | 186571486 | Intron 2 | 0.99954 | 0.1744 | T | C | 0.43207 |
| **rs3774261** | 712A>G | 186571559 | Intron 2 | 0.99991 | 0.0003 | A | G | 0.44284 |
| **rs17366743** |  | 186572089 | Exon 3 | 0.89947 | 0.7891 | T | C | 0.00357 |
| **rs6444174** |  | 186573189 | 3'-UTR | 0.99594 | 0.027 | T | C | 0.15706 |
| **rs1063537** |  | 186574075 | 3'-UTR | 0.9523 | NA | C | T | 0.04331 |
| **rs2082940** |  | 186574164 | 3'-UTR | 0.98736 | 0.0005 | C | T | 0.20264 |
| **rs1063538** | +3228 C > T | 186574183 | 3'-UTR | 0.99788 | 0.8106 | T | C | 0.44355 |
| **rs9842733** |  | 186575482 | 3'-UTR | 0.92664 | 0.5974 | A | T | 0.10153 |
| **rs1403697** |  | 186576693 | 3'-UTR | 0.90564 | 0.0028 | A | G | 0.1407 |
| **rs7641507** |  | 186578573 | 3'-UTR | 0.65977 | 0.0028 | C | T | 0.0477 |
| **rs6444175** |  | 186579744 | 3'-UTR | 0.9893 | 0.8684 | G | A | 0.31417 |
| **rs1403696** |  | 186579866 | 3'-UTR | 0.88475 | 0.5763 | C | T | 0.17523 |
| **rs13085499** |  | 186580840 | 3'-UTR | 0.8208 | 0.5096 | G | A | 0.44768 |
| **rs17373414** |  | 186585527 | 3'-UTR | 0.21543 | 0.8829 | C | T | 0.02477 |

**^(a)^** Position based on NCBI Build 36: MAF: minor allele frequency; **^(b)^**r^2^ refers to the measurement imputation quality of the SNPs. HWE: [Hardy Weinberg equilibrium](https://www.google.com/search?espv=2&biw=1280&bih=627&q=hardy+weinberg+equilibrium&spell=1&sa=X&ei=0REPVK35DI_lsATR14KwCg&ved=0CBoQvwUoAA).

S2: Association between *ADIPOQ* SNPs and adiponectin serum levels in men and women from the Jackson Heart Study cohort.

| **SNPs** |  | **Model 1** | | **Model 2** | | **Model 3** | | **Model 4** | |
| --- | --- | --- | --- | --- | --- | --- | --- | --- | --- |
|  |  |  | |  | |  | |  | |
| **rs** | **Alleles** | **β (SE)** | ***P* value** | **β (SE)** | ***P* value** | **β (SE)** | ***P* value** | **β (SE)** | ***P* value** |
| **rs4632532** | T/C | -0.054 (0.024) | 0.024 | -0.060( 0.028) | 0.033 | -0.061 (0.030) | 0.045 | -0.062 (0.026) | 0.0179 |
| **rs6444174** | T/C | 0.10 (0.025) | 0.000051 | 0.13 (0.030) | 0.000013 | 0.13 (0.032) | 0.000069 | 0.10 (0.028) | 0.00016 |
| **rs16861194** | A/G | 0.033 (0.02) | 0.10 | 0.035 (0.023) | 0.12 | 0.043 (0.025) | 0.091 | 0.045 (0.022) | 0.038 |
| **rs182052** | G/A | 0.033 (0.020) | 0.10 | 0.035 (0.023 ) | 0.13 | 0.042 (0.025) | 0.10 | 0.046 (0.022) | 0.038 |
| **rs710445** | A/G | 0.033 (0.02) | 0.096 | 0.036 (0.023) | 0.12 | 0.044 (0.025) | 0.0845 | 0.046 (0.022) | 0.034 |
| **rs16861205** | G/A | -0.123(0.031) | 0.000095 | -0.133 (0.036) | 0.00022 | -0.12 (0.039) | 0.0023 | -0.11 (0.033) | 0.00057 |
| **rs822394** | C/A | -0.071 (0.039) | 0.068 | -0.076 (0.046) | 0.097 | -0.060 (0.049) | 0.22 | -0.076 (0.043) | 0.076 |
| **rs12495941** | G/T | 0.0302(0.074) | 0.68 | -0.063 (0.098) | 0.51 | -0.16 (0.10) | 0.11 | -0.021 (0.092) | 0.81 |
| **rs7649121** | A/T | 0.049 (0.058) | 0.40 | 0.045 (0.069) | 0.51 | 0.046 (0.075) | 0.53 | 0.063 (0.065) | 0.32 |
| **rs7627128** | C/A | -0.033(0.12) | 0.78 | -0.12 (0.16) | 0.43 | -0.18 (0.17) | 0.30 | -0.146 (0.154) | 0.34 |
| **rs9877202** | A/G | -0.062 (0.036) | 0.083 | -0.060 (0.042) | 0.148 | -0.045 (0.045) | 0.31 | -0.146 (0.154) | 0.34 |
| **rs1501299** | G/T | -0.073 (0.041) | 0.075 | -0.092 (0.046) | 0.047 | -0.13 (0.050) | 0.0059 | -0.098 (0.043) | 0.024 |
| **rs3821799** | T/C | 0.039( 0.024) | 0.10 | 0.030 (0.028) | 0.28 | 0.037 (0.030) | 0.21 | 0.027 (0.026) | 0.30 |
| **rs9842733** | A/T | -0.18 (0.10) | 0.082 | -0.12 (0.12) | 0.32 | -0.093 (0.13) | 0.48 | -0.049 (0.11) | 0.66 |
| **rs1403697** | A/G | 0.091 (0.019) | 0.0000020 | 0.10 (0.022) | 0.0000018 | 0.12 (0.024) | 0.00000072 | 0.098 (0.021) | 0.0000032 |
| **rs7641507** | C/T | 0.091 (0.019) | 0.0000020 | 0.10 (0.022) | 0.0000018 | 0.12 (0.024) | 0.00000072 | 0.098 (0.021) | 0.0000032 |
| **rs1403696** | T/C | -0.069 (0.076) | 0.36 | -0.018 (0.091) | 0.84 | -0.061 (0.106) | 0.56 | 0.040 (0.085) | 0.63 |
| **rs13085499** | T/C | 0.13 (0.099) | 0.17 | 0.093 (0.12) | 0.43 | 0.053 (0.13) | 0.68 | 0.149 (0.11) | 0.18 |

Alleles listed as major /minor allele: model 1: Adjusted for BMI, age, smoking; model 2: Adjusted for age, smoking, ancestry; model 3: Adjusted for age, smoking, ancestry, BMI, income, model 4: Adjusted for Age, gender, BMI, smoking, income, PEA. The results of the association are listed as the beta (standard error) (β (SE)) with the corresponding P-value.

| **SNPs** | **Alleles** | **Normal-Weight** | | **Overweight/Obese** | |
| --- | --- | --- | --- | --- | --- |
|  |  |  | |  | |
|  |  |  | |  | |
| **rs** | **A1/A2** | **β (SE)** | ***P* value** | **β (SE)** | ***P* value** |
| **rs4632532** | T/C | -0.0117 (0.010) | 0.2584 | -0.0014 (0.0097) | 0.8879 |
| **rs~~644~~4174** | T/C | 0.0418 (0.0113) | 0.0002839 | 0.0021 (0.0102 | 0.8343 |
| **rs16861194** | A/G | 0.0176 (0.0083) | 0.03528 | -0.0014 (0.0081) | 0.8648 |
| **rs182052** | G/A | 0.0162 (0.0083) | 0.05361 | -0.0006 (0.0081) | 0.9436 |
| **rs710445** | A/G | 0.0175 (0.0083) | 0.035 | -0.0016 (0.0080) | 0.8458 |
| **rs16861205** | G/A | 0.0295 (0.0126) | 0.0194 | 0.0105 (0.0125) | 0.4004 |
| **rs822394** | C/A | -0.0301 (0.0161) | 0.06228 | 0.0044 (0.0155) | 0.7788 |
| **rs12495941** | G/T | -0.0024 (0.0281) | 0.9309 | -0.0009 (0.0347) | 0.9793 |
| **rs7649121** | A/T | -0.0127 (0.0239) | 0.5959 | -0.0183 (0.0236) | 0.4381 |
| **rs7627128** | C/A | -0.0474 (0.0503) | 0.3473 | -0.0102 (0.0568) | 0.8573 |
| **rs9877202** | A/G | -0.0256 (0.0145) | 0.07859 | 0.0027 (0.0142) | 0.847 |
| **rs1501299** | G/T | 0.0045 (0.0164) | 0.7829 | -0.0249 (0.0159) | 0.1174 |
| **rs3821799** | T/C | 0.0115 (0.0104) | 0.2727 | 0.0085 (0.0095) | 0.3729 |
| **rs9842733** | A/T | 0.0809 (0.0357) | 0.02435 | -0.0245 (0.0446) | 0.5832 |
| **rs1403697** | A/G | -0.0027 (0.0082) | 0.7388 | -0.0000 (0.0078) | 0.995 |
| **rs7641507** | C/T | -0.0027 (0.0082) | 0.7412 | -0.0000 (0.0078) | 0.9984 |
| **rs13085499** | G/A | 0.0724 (0.0387) | 0.06262 | -0.0705 (0.0419) | 0.09261 |

S3: Association between *ADIPOQ* SNPs and BMI in normal weight and overweight or obese men and women from the Jackson Heart Study cohort.

Alleles listed as major/minor allele; Adjusted for sex, age, smoking, PEA and income. The results of the association are listed as the beta (standard error) (β (SE)) with the corresponding P-value. β Coefficients represent the change in absolute traits values of each additional risk allele.
